# Glutathione catabolism by *Enterobacteriaceae* species to hydrogen sulphide adversely affects the viability of host systems in the presence of 5′fluorodeoxyuridine

**DOI:** 10.1111/mmi.14893

**Published:** 2022-03-22

**Authors:** Daniel Rui Xiang Lim, Yahua Chen, Li Fang Ng, Jan Gruber, Yunn‐Hwen Gan

**Affiliations:** ^1^ Department of Biochemistry, Yong Loo Lin School of Medicine National University of Singapore Singapore Singapore; ^2^ Science Divisions Yale, NUS College Singapore Singapore; ^3^ Infectious Diseases Translational Research Programme, Yong Loo Lin School of Medicine National University of Singapore Singapore Singapore

**Keywords:** bacteria, *Caenorhabditis elegans*, glutathione, hydrogen sulphide, intestine

## Abstract

Reduced glutathione (GSH) plays an essential role in relieving oxidative insult from the generation of free radicals via normal physiological processes. However, GSH can be exploited by bacteria as a signalling molecule for the regulation of virulence. We describe findings arising from a serendipitous observation that when GSH and *Escherichia coli* were incubated with 5′fluorodeoxyuridine (FUdR)‐synchronised populations of *Caenorhabditis elegans*, the nematodes underwent rapid death. Death was mediated by the production of hydrogen sulphide mainly through the action of *tnaA*, a tryptophanase‐encoding gene in *E. coli*. Other Enterobacteriaceae species possess similar cysteine desulfhydrases that can catabolise l‐cysteine‐containing compounds to hydrogen sulphide and mediate nematode killing when worms had been pre‐treated with FUdR. When colonic epithelial cell lines were infected, hydrogen sulphide produced by these bacteria in the presence of GSH was also able to inhibit ATP synthesis in these cells particularly when cells had been treated with FUdR. Therefore, bacterial production of hydrogen sulphide could act in concert with a commonly used genotoxic cancer drug to exert host cell impairment. Hydrogen sulphide also increases bacterial adhesion to the intestinal cells. These findings could have implications for patients undergoing chemotherapy using FUdR analogues that could result in intestinal damage.

## Graphical abstract

Enterobacterial species found in the gastrointestinal tract have the ability to convert antioxidant glutathione to hydrogen sulfide, which in concert with chemotherapeutic drug 5 fluorodeoxyuridine (FUdR), can cause rapid death in *Caenorhabditis elegans*. In mammalian intestinal cells, exposure to hydrogen sulfide and FUdR cause cells to lose viability with decrease in ATP production. These findings could have implications for patients undergoing chemotherapy using FUdR analogues that could result in intestinal damage.

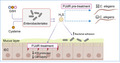

## 1 INTRODUCTION

Reduced glutathione (GSH) is a low molecular weight thiol that is a major component of the cytosol. A tri‐peptide consisting of glycine, glutamate and cysteine, GSH is present abundantly in most eukaryotes as well as some prokaryotes such as cyanobacteria and proteobacteria (Masip et al., 2006). Its major physiological roles include detoxification of reactive oxygen species (ROS) as well as a post‐translational protein modifier as part of S‐glutathionylation (Xiong et al., 2011). Higher GSH concentrations are often associated with reduced oxidative stress while in many disease conditions such as diabetes mellitus or HIV infection, low GSH concentrations are correlated with poorer survival and increased morbidity (Dinçer et al., 2002; Herzenberg et al., 1997).

More recently, GSH has been shown to be exploited by bacteria as a host signal to turn on virulence. *Listeria monocytogenes*, the causative agent of Listeriosis, uses GSH as a signal to turn on its master regulator of virulence, PrfA (Reniere et al., 2015). In *Burkholderia pseudomallei*, the causative agent of melioidosis, GSH has been shown to activate the Type VI secretion system (T6SS) that is used for cell fusion as a means of bacterial intracellular spread (Wong et al., 2015). Other deleterious effects of GSH stem from its ability to form toxic compounds, such as hydrogen sulphide (Chu, 2002) due to the cysteine content of GSH.

Therefore, we wanted to determine if there are other effects that GSH could exert on clinically relevant bacteria that could impact their pathogenesis. Besides being abundant in the cytoplasm of eukaryotic cells, GSH is also abundant in the human gastrointestinal (GI) lumen, a reducing and anaerobic environment where glutathione remains in the reduced form (Circu & Aw, 2011). In addition, the human body contains large microbial communities in the GI tract (Thomas et al., 2017) and the effects of GSH in the context of virulence enhancement of possible pathobionts in the GI tract have not been well‐studied.

In this article, we describe an interaction between GSH, *Caenorhabditis elegans* and several species of the Enterobacteriaceae under the influence of 5′fluorodeoxyuridine (FUdR) that leads to rapid *C. elegans* death. We examine and discuss the possible relevance to mammalian cells and the implications during colorectal cancer treatment.

## 2 RESULTS

### 2.1 GSH induces rapid death of FUdR‐treated *C. elegans* in the presence of *Escherichia coli* OP50

**FIGURE 1:**
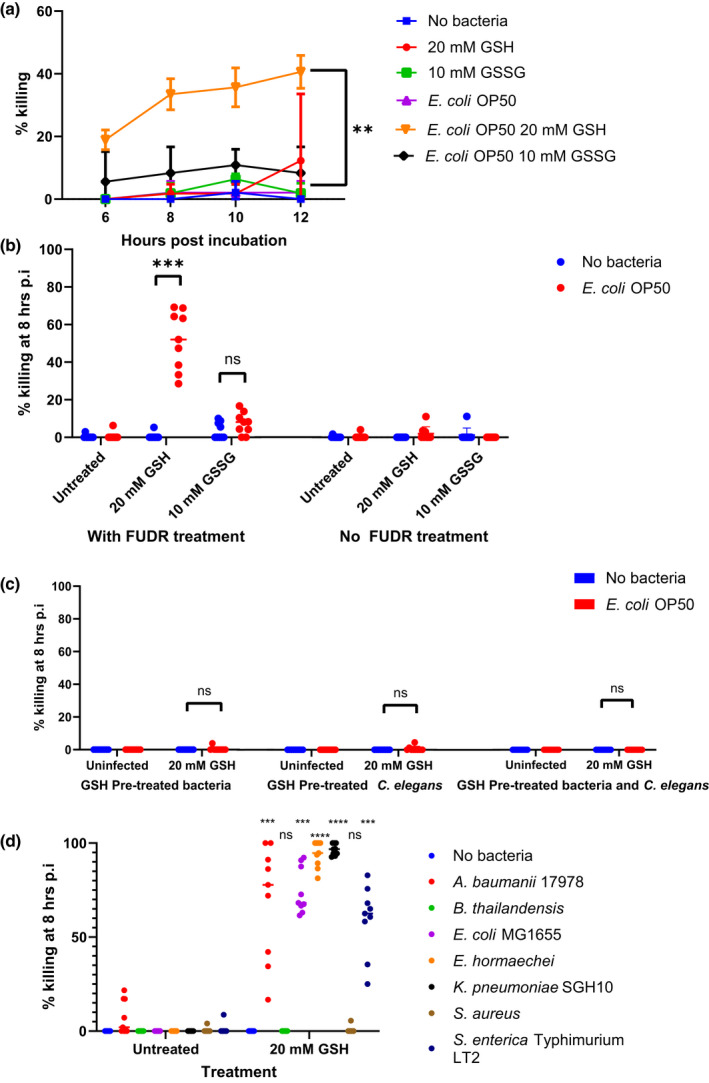
Live bacteria induce the rapid death of FUdR‐treated *Caenorhabditis elegans* in the presence of GSH. (a) 300 μM FUdR‐synchronised adult *C. elegans* were incubated with 20 mM GSH or 10 mM GSSG and live *Escherichia coli* OP50. Percentage death was calculated at each timepoint between 6 and 12 h. Each point represents the means ± SD (*n* = 3). One‐way ANOVA was used to compare means of each treatment group after confirming that data followed a normal distribution using Shapiro–Wilk’s test. Dunnett’s post hoc test was then used to find significantly different treatment groups from the control group. (b) Adult *C. elegans* synchronised using 300 μM FUdR or not for 24 h were incubated with 20 mM GSH or 10 mM GSSG and live *E. coli* OP50. Percentage killing was calculated at 8 h postincubation. A horizontal line represents the means ± SD of three independent experiments (*n* = 3). Student’s *t*‐test was used to compare means of treated groups to that of the untreated control, ***p < .001 ns, not significant. (c) *C. elegans* were pre‐treated with 20 mM GSH or 10 mM GSSG for 4 h before washing to remove GSH and then incubated with *E. coli* OP50. Pre‐treatment of *E. coli* OP50 with 20 mM of GSH (left panel), *C. elegans* (middle panel), or both *C. elegans* and *E. coli* OP50 (right panel) for 4 h before washing to remove GSH was carried out before incubation with bacteria or *C. elegans*. Percentage killing was calculated at 6 h postincubation. A horizontal line represents the means ± SD of three independent experiments (*n* = 3). (d) FUdR‐treated adult *C. elegans* were incubated with *Acinetobacter baumannii* 17978, *Burkholderia thailandensis* E264, *Enterobacter hormaechei*, *E. coli* MG1655, *Klebsiella pneumoniae* SGH10, *Staphylococcus aureus* or *Salmonella enterica Typhimurium* LT2 in the presence or absence of 20 mM GSH. Percentage killing was calculated at 8 h postincubation for both treatment groups a horizontal line represents the means ± SD of three independent experiments (*n* = 3) *p < .05. Student’s *t*‐test was used for comparison between treatment and control groups

To screen for potential bacterial virulence enhancing properties of GSH, we used *C. elegans* as a surrogate host model. This model has frequently been used for screening of bacterial virulence (Sifri et al., 2005). We discovered that in the control group where live *C. elegans* food source *E. coli* OP50 was added together with 20 mM GSH, a significant number of *C. elegans* died rapidly in 6–12 h (Figure 1a). This happened regardless of whether the bacteria were from stationary or log‐phase culture (Figure S1). The killing was mediated by reduced glutathione (GSH), but not oxidised glutathione (GSSG).

However, the killing depended on the *C. elegans* being treated with FUdR before the assay. FUdR is a metabolite of 5‐fluorouracil (5‐FU) used in the treatment of many gastrointestinal cancers (Miura et al., 2010; Tang et al., 2016). It is an anti‐metabolite that inhibits DNA synthesis and hence, cell growth, resulting in life cycle arrest. In experiments with *C. elegans*, nematodes are routinely treated with FUdR to prevent progeny production. The viability of adult *C. elegans* are generally not affected by FUdR as their somatic cells do not reproduce (Sulston et al., 1983), and hence have no need to replicate DNA.

When *C. elegans* that had not been treated with FUdR were incubated with *E. coli* OP50 and GSH, we did not observe the rapid‐killing phenotype (Figure 1b). It should be noted that FUdR was not present during the killing assay. We had found that L4 and adult *C. elegans* shared the same susceptibility to killing, Therefore, adult *C. elegans* were used for subsequent experiments for ease of counting and for consistency. We also examined the effect of FUdR and GSH on sterile mutant worms JK1107 to decipher whether the effect of FUdR was dependent on the worms being made sterile. Our results showed that the rapid killing was completely abolished in the mutant worms treated with GSH and bacteria, whether in the presence or absence of FUdR (Figure S2). This suggests that toxicity is dependent on the effect of FUdR on germline cells, which are absent in the mutants at the temperature the experiment was conducted.

### 2.2 Pre‐treatment with GSH of bacteria or *C. elegans* does not predispose *C. elegans* to rapid death

To determine whether GSH itself was acting as a signal to bacteria to turn on expression of virulence genes that resulted in the killing observed, or if GSH was acting on the host and predisposing *C. elegans* to bacterial killing, we tested whether pre‐treatment of either *C. elegans* or bacteria with GSH would result in the rapid killing.

In the first scenario, we treated bacteria with 20 mM of GSH or 10 mM of GSSG for 4 h before removing GSH and then incubating it with *C. elegans* for eight more hours. The second scenario involved *C. elegans* treated with the same concentrations of GSH or GSSG for 4 h before incubation with bacteria for eight more hours. Lastly, both *C. elegans* and bacteria were also separately pre‐treated for 4 h before they were incubated together for another 8 h. Rapid killing of nematodes was not observed in these cases, strongly suggesting that GSH had to be present during the course of the assay for rapid death to be induced (Figure 1c).

To examine whether this phenomenon can be observed in other types of bacteria, we included several other species for comparison. Of the various bacterial species tested, the killing was most pronounced when GSH was incubated with members of the Enterobacteriaceae (Figure 1d), a large family of Proteobacteria that include many clinically relevant species that are nosocomial and opportunistic. We also included Gram‐negative *Acinetobacter baumannii* that belongs to the *Moraxellaceae* family for comparison.

As with avirulent *E. coli* OP50, the *E. coli* K12 laboratory‐adapted strain MG1655 could be induced to cause rapid killing of *C. elegans*. In fact, the killing was generally higher than that of OP50. This rapid‐killing of *C. elegans* by normally avirulent bacteria suggests that the mechanism of killing is quite different from bacterial virulence factors such as those seen in *Salmonella typhimurium* and *Staphylococcus aureus*, which typically kill *C. elegans* over a period of days (Aballay et al., 2000; Sifri et al., 2003). The only two species tested here that did not kill the nematodes within this time period are *B. thailandensis* and Gram‐positive *S. aureus*. *B. thailandensis* was previously shown to kill *C. elegans* over 2–3 days (Gamage et al., 2018).

One possibility was that GSH altered the internal cellular redox balance thereby potentiating *C. elegans*’ death when challenged with bacteria. However, testing with other reducing agents, including DTT, which is thiol‐based similar to GSH, and tris(2‐carboxyethyl)phosphine (TCEP) which is not thiol‐based, did not induce death (Figure S3).

### 2.3 GSH is catabolised to form hydrogen sulphide (H_2_S) by tryptophanase in *E. coli*

**FIGURE 2:**
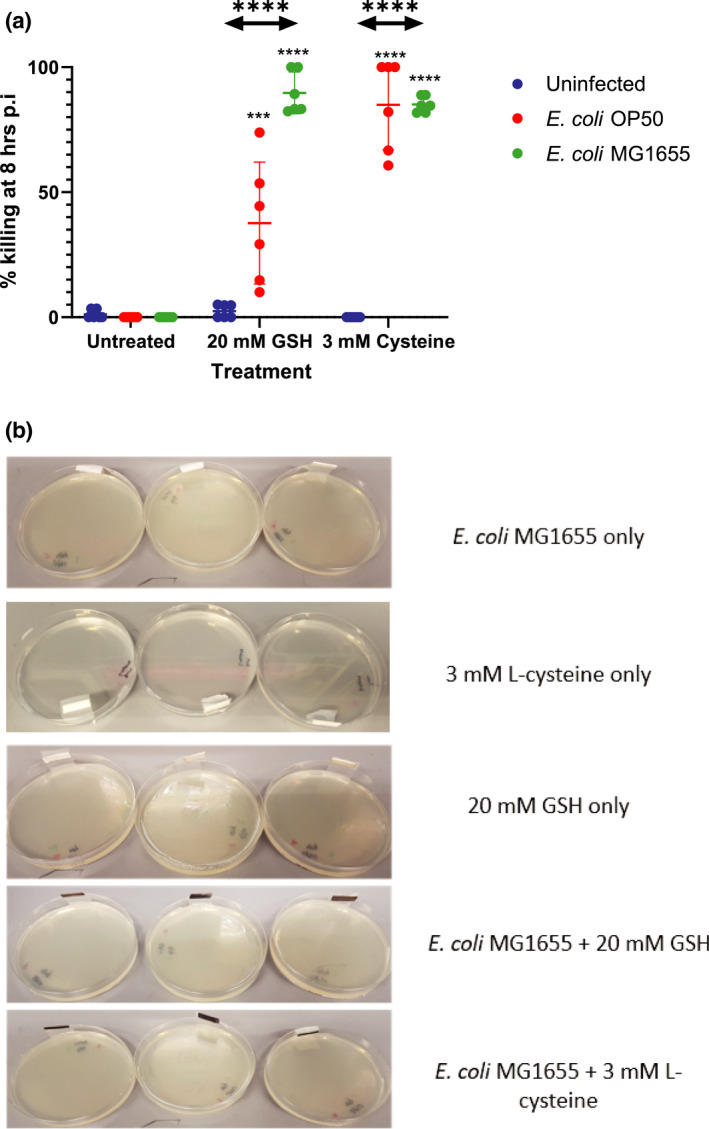
Cysteine and GSH catabolism to H_2_S is responsible for the rapid killing in *Caenorhabditis elegans*. (a) FUdR‐treated *C. elegans* were incubated with *Escherichia coli* strains OP50 or MG1655 in the presence of 3 mM l‐cysteine or 20 mM GSH. Percentage killing was calculated at 8 h postincubation and compared to the untreated control using Student’s *t*‐test. One‐way ANOVA was used to compare means within each treatment group after confirming that data followed a normal distribution using Shapiro–Wilk’s test. Dunnett’s post hoc test was then used to find significantly different groups. A horizontal line represents the means ± SD of two independent experiments (*n* = 3), *** *p* < .001, ns, not significant. (b) *E. coli* MG1655 was incubated on nutrient agar with 20 mM GSH or 3 mM cysteine at 20°C. Lead acetate strips were attached to the side of each petri dish and examined after 24 h to detect the production of hydrogen sulphide if the strip turns black. The experiment was conducted with triplicate plates and repeated three times with similar results

We then examined the possibility of the generation of a quick‐acting toxic substance. A previous report showed that GSH could be used as a substrate for H_2_S formation through an unknown pathway involving cystalysin in *Treponema denticola* (Chu, 2002). This pathway is likely similar to the one that cysteine undergoes in its catabolism to H_2_S. Indeed, we verified that l‐cysteine had the same killing effect as GSH (Figure 2a). When *E. coli* was tested for H_2_S production with GSH and l‐cysteine, a colour change in lead acetate strips from white to black confirmed that H_2_S was produced (Figure 2b).

### 2.4 Bacterial sulphide production is correlated with rapid killing

We decided to identify homologues of sulphide‐producing enzymes in *E. coli* MG1655 (Table 1) to determine if deleting them would abolish rapid killing. One such candidate, TnaA, is a tryptophanase possessing cysteine desulfhydrase activity in *E. coli* MG1655. It was reported that *tnaA* is highly inducible by the addition of l‐cysteine (Awano et al., 2005). TnaA comprises up to 10% of soluble protein in *E. coli* (Snell, 2006), and can degrade l‐cysteine to hydrogen sulphide, ammonia and pyruvate.

**TABLE 1:** Presence of H_2_S‐producing enzyme homologueshomologs in various bacteria species

Bacteria species	Cysteine desulfhydrase	Cystathionine‐γ‐lyase	3‐Mercaptopyruvate sulfurtransferase	Rapid killing of *Caenorhabditis elegans*
*A. baumanii*	*dcyD* (for d‐cysteine)	–	*sseA*	Yes
*B. thailandensis* E264	–	–	–	No
*E. coli*	*tnaA* (Awano et al., 2005)	*metC*	*mstA*	Yes
*E. cloacae*	*tnaA*	*metC*	*sseA*	Yes
*K. pneumoniae*	–	*metC* (Seiflein & Lawrence, 2006)	*sseA*	Yes
*S. aureus*	–	*metC*	–	No
*S. enterica* Typhimurium LT2	*cdsH* (Oguri et al., 2012)	–	*sseA*	Yes

**FIGURE 3:**
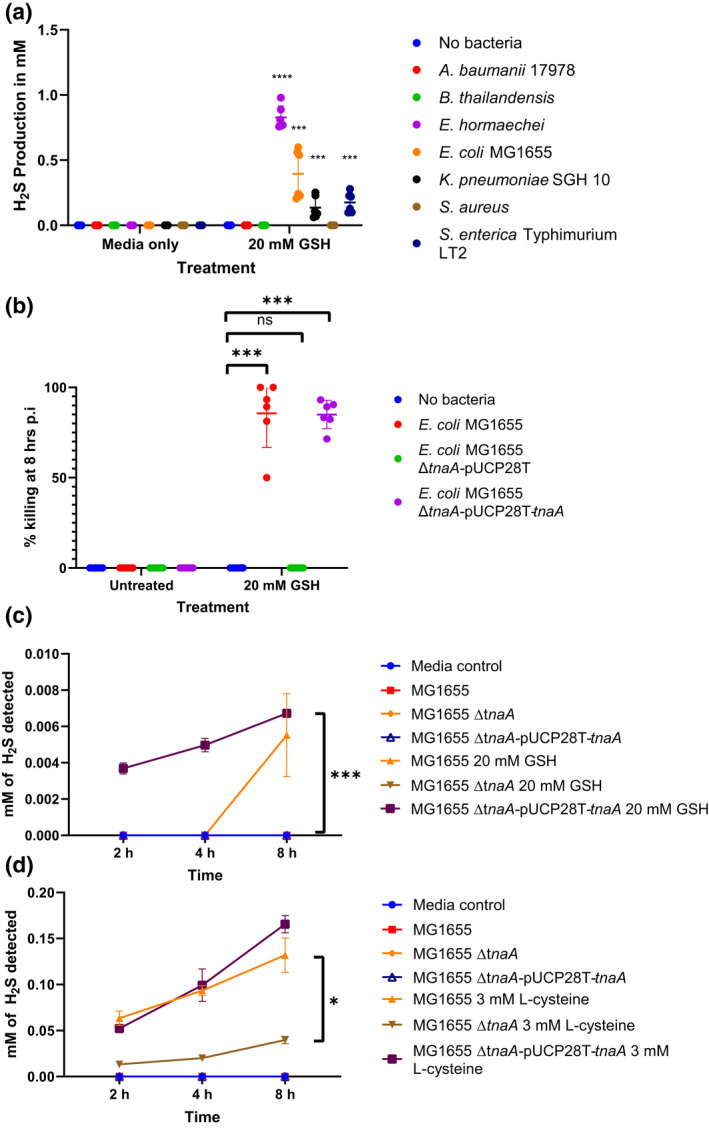
H_2_S production is mediated by tryptophanase in *Escherichia coli*. (a) Various bacterial strains were assessed for their ability to convert 20 mM GSH to H_2_S using a methylene blue assay. Absorbance values were converted to H_2_S concentrations by comparison with the Na_2_S standard curve over 24 h. The experiment was carried out twice (*n* = 3). A horizontal line represents the mean ± SD of two independent experiments (*n* = 3). Student’s *t*‐test values were obtained by comparing the mean of bacteria with GSH treatment groups to their untreated controls, ***p < .001, ns, not statistically significant. (b) a Δ*tnaA E. coli* MG1655 strain and its complement were assessed for its ability to rapidly kill adult FUdR‐treated *Caenorhabditis elegans* with 20 mM GSH. A horizontal line represents the mean ± SD of two independent experiments (*n* = 2). (b) a Δ*tnaA E. coli* MG1655 strain and its complement were assessed for its ability to rapidly kill adult FUdR‐treated *C. elegans* with 20 mM GSH. A horizontal line represents the mean ± SD of two independent experiments. (*n* = 2). One‐way ANOVA was used to compare means within GSH‐treated groups after confirming that data followed a normal distribution using Shapiro–Wilk’s normality test. Dunnett’s post hoc test was then used to find significantly different treatment groups from the GSH‐only control. (c, d) A time‐course methylene blue H_2_S production assay of *E. coli* MG1655 and its Δ*tnaA* mutant with 20 mM GSH (c) and 3 mM l‐cysteine (d) over 8 h was carried out to assess the kinetics of GSH and l‐cysteine catabolism. A horizontal line represents the mean ± SD (*n* = 3) the experiment was repeated three times independently. Student’s *t*‐test values were obtained by comparing the mean of bacteria with GSH treatment groups to their untreated controls. *p < .05, ***p < .001 ns, not significant

We then examined H_2_S production by various Enterobacteriaceae species. While most of them could produce H_2_S from GSH, they did not do so with the same efficiency (Figure 3a). *E. hormaechei* for instance, produced fourfold as much H_2_S as *E. coli* MG1655 over a 24 h period. The surprising outlier was *A. baumanii* 17978, which showed rapid killing under GSH stimulation (Figure 1d) but did not produce detectable sulphide (Figure 3a).

To determine whether H_2_S production by TnaA was responsible for the rapid killing, we deleted *tnaA* in *E. coli* MG1655. The mutant strain of MG1655 no longer induced rapid killing. Complementation of the Δ*tnaA* strain with the original gene on the pUCP28T plasmid vector, however, restored the rapid killing of *C. elegans*, whereas complementation with just the plasmid vector did not (Figure 3b).

### 2.5 TnaA is responsible for the majority of GSH catabolism to H_2_S

Over the course of 24 h, measurement of sulphide concentrations through catabolism of GSH (Figure 3c) and cysteine (Figure 3d) using a methylene blue assay showed that the amount of hydrogen sulphide produced by wild‐type and TnaA complemented‐*E. coli* MG1655 with 20 mM GSH was 10 μM. This was tenfold lower when compared to l‐cysteine, due to the extra steps needed to convert GSH to its component amino acids.

It is also likely that our assay could only capture a fraction of the sulphide produced from the catabolism of GSH or l‐cysteine since only gaseous H_2_S diffuses out from the inner petri dish containing the bacteria‐GSH/l‐cysteine solution is captured for measurement.

When a time‐course experiment was carried out over 8 h with 20 mM GSH and 3 mM l‐cysteine, we observed that H_2_S production by 8 h (Figure 3c,d) was sufficiently high to be above the physiological norm of nanomolar H_2_S tissue concentrations (Furne et al., 2008). The concentration of H_2_S produced by the Δ*tnaA* mutant with l‐cysteine was less than half of wild‐type (Figure 3d) while the complemented Δ*tnaA* mutant was able to produce H_2_S at an efficacy similar to that of wild‐type. The kinetics of H_2_S production also correlated with *C. elegans* killing, where death would be observed by 8 h post‐addition of bacteria and GSH or l‐cysteine.

### 2.6 Toxic effects of FUdR and H_2_S on mammalian cells

**FIGURE 4:**
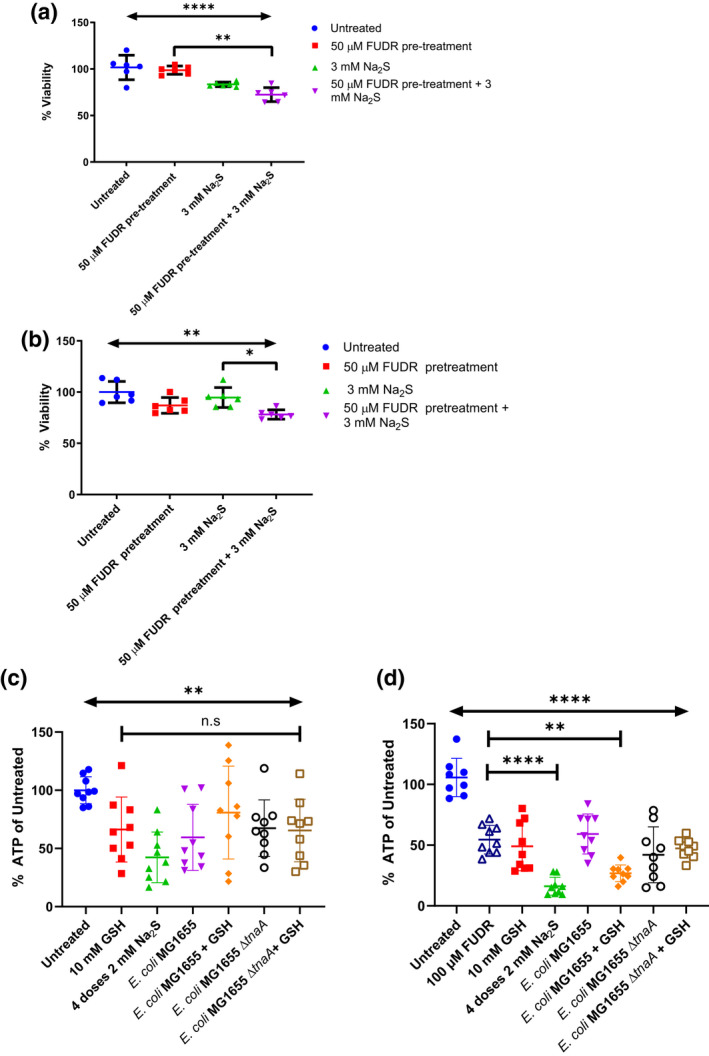
FUdR and H_2_S exhibit greater toxicity in combination to mammalian cells. (a) XTT viability assay of Caco‐2 cells when pre‐treated with 50 μM of FUdR and two doses of 3 mM H_2_S‐releasing Na_2_S (fourth group from left) 2 h apart compared to mono‐treatment of FUdR (second bar from left) or three two doses of 3 mM Na_2_S 2 h apart for a total of 4 h (third group from left). A horizontal line represents the mean ± SD of two independent experiments (*n* = 2). One‐way ANOVA was used to compare the means of all groups to that of the FUdR and Na_2_S combined treatment group after confirming that data followed a normal distribution using Shapiro–Wilk’s test. Dunnett’s post hoc test was then used to find significantly different treatment groups. (b) XTT viability assay of HT‐29 MTX P8 cells pre‐treated with 50 μM of FUdR for 24 h before washing to remove it followed by addition of two doses of 3 mM Na_2_S 2 h apart for a total of 4 h. (a) Horizontal line represents the mean ± SD of two independent experiments (*n* = 2). One‐way ANOVA was used to compare the means of all groups to that of the FUdR and Na_2_S combined treatment group after confirming that data followed a normal distribution using Shapiro–Wilk’s test. Dunnett’s post hoc test was then used to find significantly different treatment groups. (c) 2 × 10^5^ cells were seeded in a 24‐well plate with 10 μM DAPT for 3 days and subsequently incubated with various *Escherichia coli* MG1655 strains at an MOI of 50 with or without GSH for 4 h before measurement of intracellular ATP. A horizontal line represents the mean ± SD of three independent experiments (*n* = 3). One‐way ANOVA was used to compare the means between untreated and treatment groups, as well as within treated groups after confirming that data followed a normal distribution using the Anderson–Darling normality test. (d) 2 × 10^5^ cells were seeded in a 24‐well plate with 10 μM DAPT for 3 days as well as 100 μM of FUdR on the last day. They were then subsequently incubated with various *E. coli* MG1655 strains at an MOI of 50 with or without GSH for 4 h before measurement of intracellular ATP. A horizontal line represents mean ± SD of three independent experiments (*n* = 3). One‐way ANOVA was used to compare the means of FUdR‐treated groups (all groups except untreated) to the FUdR‐only group after confirming that data followed a normal distribution using the Anderson–Darling normality test. Dunnett’s post hoc test was then used to find significantly different treatment groups, **p < .01, ****p < .0001, ns, not significant

To determine whether the toxic effects of H_2_S on FUdR‐treated *C. elegans* could be observed in mammalian cell lines, we examined Caco‐2 cells pre‐treated with FUdR and two doses of 3 mM Na_2_S, which causes a rapid release of H_2_S (Ng et al., 1993) over 1 hour. We used Na_2_S as a chemical sulphide donor in the XTT viability assay as GSH interferes with the downstream formation of coloured formazan. We observed a greater reduction in cell viability in the wells co‐treated with both compounds compared to individually treated cells for both Caco‐2 (Figure 4a) and HT29 MTX P8 (Figure 4b) cell lines. However, these decreases appeared additive and were very different from what we observed with *C. elegans*, where there was a rapid killing phenotype only when the two treatments were administered, and none observed with either FUdR or GSH in isolation.

To measure if cells were significantly impacted by the bacteria‐GSH treatment, we examined intracellular ATP concentrations in FUdR‐untreated (Figure 4c) and FUdR pre‐treated HT‐29 MTX P8 cells (Figure 4d) that were incubated with either bacteria only or bacteria with GSH added and compared these to positive control groups that were treated with Na_2_S. A lower concentration of 100 μM FUdR was used as compared to that used for *C. elegans* because we expected mammalian cells, having to undergo cell division, would be more sensitive to the effects of FUdR. This FUdR concentration was also the highest we could use without any significant impact on cell viability.

Intracellular ATP concentrations were reduced in all compared to the untreated control. Nevertheless, it was striking that under FUdR treatment, wild‐type *E. coli* MG1655 that was able to catabolise GSH to H_2_S caused a significant reduction in intracellular ATP concentration in the cells compared to the FUdR‐treated control only when GSH was added with the *E. coli*, whereas cells incubated with the Δ*tnaA* deletion mutant and GSH did not exhibit a significant decrease in ATP compared to the Δ*tnaA* bacteria only group. These differences show that the TnaA‐catalysed breakdown of GSH to H_2_S greatly aggravates the drop in ATP production similar to the effect of Na_2_S treatment alone. This reduction was not seen in cells that were not pre‐treated with FUdR. Further evidence that sulphide is responsible is shown by the fact that in the *E. coli* Δ*tnaA* mutant, the addition of GSH did not cause a drop in ATP concentrations, unlike the wild‐type bacteria with GSH. Without FUdR treatment, however, the differences between these groups disappeared.

These differences also occurred without a significant difference in cell count between the various treatment groups (Figure S4), which indicated that the likely cause for the differences in intracellular ATP concentrations was due to the effects of sulphide and FUdR on cellular metabolism.

### 2.7 H_2_S enhances adhesion of bacteria to epithelial cells

To determine if the H_2_S produced could have other effects on bacterial and host cell interactions, we pre‐ or co‐treated HT‐29 MTX P8 colorectal epithelial cells with GSH or Na_2_S to examine whether bacterial adhesion could be enhanced by this treatment.

**FIGURE 5:**
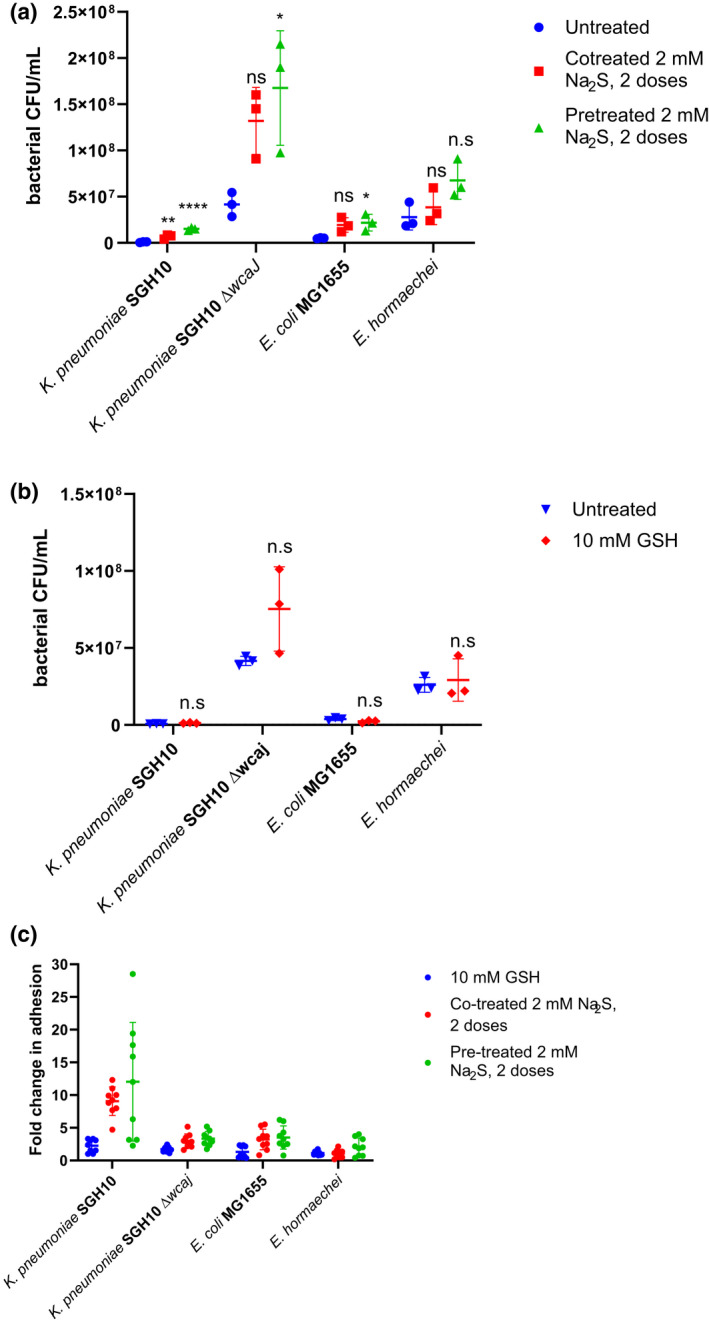
Sulphide treatment enhances bacterial adhesion to intestinal epithelial cell lines. (a, b) HT‐29 MTX P8 cells were infected at an MOI of 50 with a 2 h pre‐treatment or co‐treatment of two doses of 2 mM Na_2_S before bacterial incubation. One‐way ANOVA was used to compare the means within each bacterial group after confirming that data followed a normal distribution using Shapiro–Wilk’s normality test. Dunnett’s post hoc test was then used to find significantly different treatment groups from the untreated group. *p < .5, **p < .01, ***p < .001 and ns, not significant. (a) or 10 mM of GSH (b). The monolayers were then washed and treated with 0.25% triton‐X for cell lysis before the supernatant was diluted and plated on LB media to enumerate CFU of adhered bacteria. A horizontal line represents mean ± SD of three independent experiments (*n* = 3). Student’s *t*‐test was used to compare the means of the GSH‐treatment group to that of the untreated control. NS, not significant. (c) A summary of the various replicates from (a) and (b) is presented by comparing the fold change in bacterial adhesion at 2 h with 10 mM GSH, co‐treatment of bacteria with two doses of 2 mM Na_2_S or a pre‐treatment with two doses of 2 mM Na_2_S before washing to remove Na_2_S to that of the untreated controls. A horizontal line represents mean ± SD of three independent experiments (*n* = 3)

We compared *E. coli* MG1655, hypervirulent *K. pneumoniae* SGH10 as well as *E. hormaechei* ATCC 70323 as the latter was found to produce the highest amount of H_2_S with GSH. Bacterial adhesion was significantly enhanced over untreated controls in the presence of Na_2_S (Figure 5a) but not GSH (Figure 5b). This could indicate that the H_2_S produced from GSH catabolism on its own may not be sufficient in terms of concentration or duration for the enhancement of bacterial adhesion, as it dissipates rapidly and is detoxified by colonic epithelial cells efficiently (Mimoun et al., 2012).

With sufficient H_2_S provided by the repeated administration of 2 mM Na_2_S, however, the increase in bacterial adhesion was significant. In particular, for hypermucoid *K. pneumoniae* SGH10 which generally adhered poorly to epithelial cells (Tan et al., 2020), Na_2_S pre‐and co‐treatment was able to significantly increase its adhesion by an average of 10 fold (Figure 5a,c). In a capsule‐null mutant SGH10Δ*wcaj* which has the initial glycosyltransferase deleted to prevent capsule formation, the increase in adhesion by sulphide treatment was more modest at 3‐ to 4‐fold, similar to that seen in *E. coli* MG1655. However, this enhancement was not statistically significant for *E. hormaechei*, which was very efficient at producing H_2_S from GSH and l‐cysteine (Figure 5a,c). Part of the issue could be that *E. hormaechei* is susceptible to the presence of GSH or sulphide as can be seen in the reduced growth starting from early time points (Figure S5). This could have reduced the effect on adhesion, although the trend of increased adhesion was still observed, particularly when cells were pre‐treated with H_2_S, and would have less of a direct effect on the bacteria.

## 3 DISCUSSION

GSH is best known for its role as an antioxidant, undergoing reversible oxidation to GSSG for maintenance of a constant internal redox environment. However, GSH can be sensed and exploited by cytosolic bacterial pathogens as a signal to upregulate virulence mechanisms once they have invaded a cell such as *B. pseudomallei* (Wong et al., 2015) and *L. monocytogenes* (Reniere et al., 2015).

A report has also linked cysteine to the regulation of virulence in enterohemorrhagic *E. coli* (EHEC) (Pifer et al., 2018). In EHEC, cysteine binds to the cysteine‐responsive transcription factor CutR that enhances the expression of FadL. FadL represses the inhibition of virulence expression by FadR and activates the type III secretion system governed by the locus of enterocyte effacement. It is possible that other bacterial species possess similar mechanisms for switching on their virulence mechanisms, given the abundance of GSH and cysteine in the human body (Meister, 1988).

Bacterial catabolism of l‐cysteine to sulphide has been reported to be toxic to *C. elegans* (Livshits et al., 2017). The authors found that 3 mM of l‐cysteine could produce enough hydrogen sulphide to cause nearly complete killing of *C. elegans* exposed to it using an inverted plate assay. The authors utilised synchronised nematodes and might not have considered the importance of FUdR in the process. We discovered that GSH could likewise undergo catabolism to form toxic sulphide and this killed nematodes pre‐treated with FUdR. This explains why the laboratory food source of *C. elegans*, *E. coli* OP50 could be killing its predator with GSH stimulation. We subsequently switched to using *E. coli* MG1655, a K‐12 laboratory strain due to its ease of genetic manipulation and better genetic characterisation.

We found that GSH can be catabolised into toxic H_2_S by TnaA, a tryptophanase ubiquitous in *E. coli* (Awano et al., 2005) that also has cysteine desulfhydrase activity. However, the ability to produce enough sulphide in a short amount of time is likely to be as important as the ability to produce sulphide continuously for induction of killing. Although we did not observe any killing by the *tnaA* deletion mutant at the 8 h timepoint, it is possible that given enough time, death of *C. elegans* would occur as well under GSH stimulation since H_2_S production in the deletion mutant with l‐cysteine was not completely abolished and was still increasing from 8 to 24 h postincubation.

Under the physiological concentrations of millimolar GSH, *E. coli* MG1655 is capable of converting GSH to H_2_S at concentrations of about 0.2 mM after 24 h. This is much higher than the physiological nanomolar concentrations at which it is present in tissue (Furne et al., 2008). We also examined the presence of TnaA and other hydrogen sulphide‐producing enzymes in those bacterial species (Table 1) and found that there is good correlation between the ability to produce H_2_S and killing of *C. elegans*. The case of *A. baumanii* killing under GSH stimulation may not have anything to do with sulphide production, as none was detected with methylene blue. A previous report (Rumbo‐Feal et al., 2017) provided useful clues as to the underlying mechanism behind *A. baumanii* 17978 GSH‐induced killing. A1S_0114 is a gene responsible for the production of Ac‐505, a three‐amino acid lipopeptide of *A. baumanii* that is linked to its virulence. In mutants of A1S_0114, *C. elegans* produced significantly less progeny. Crucially, GSH modification of a precursor seems to be required for the production of Ac‐505. This would explain the result that killing by *A. baumanii* was independent of sulphide production yet required the presence of GSH.

The ability to rapidly produce detectable H_2_S among the strains we tested was mostly restricted to *Enterobacteriales* species. This probably reflects the adaptations by these species to their usual niche in the human body. The gut contains high levels of GSH and cysteine in a reducing environment and is also where many Enterobacteriaceae species reside, usually comprising close to 1% of the healthy gut microbiota (The Human Microbiome Project Consortium et al., 2012). Hence, the ability to produce high concentrations of H_2_S could be a way to kill off their competitors and overcome colonisation resistance (Dordević et al., 2020).

The toxic effects of H_2_S stem from its ability to inhibit cytochrome c oxidase and thereby shutting down oxidative phosphorylation (Jiang et al., 2016). Resistance to sulphide toxicity, however, varies greatly by cell type, especially for those which normally encounter H_2_S in their environment. It has been reported that neurons are most sensitive to sulphide inhibition of oxidative phosphorylation, while epithelial cells, particularly those in the gastrointestinal tract, show high resistance to sulphide (Lagoutte et al., 2010).

Other reports have linked H_2_S to genotoxicity, likely due to its ability to generate free radicals from dysregulation of oxidative phosphorylation that may lead to DNA damage (Hoffman et al., 2012). This is corroborated by higher activity levels of DNA repair enzymes observed when intestinal cells were exposed to sulphide (Attene‐Ramos et al., 2010). One postulate is that the combined activity of FUdR inhibiting accurate DNA synthesis and sulphide acting as a genotoxic agent could cause severe genotoxic stress and lead to significantly decreased somatic cell viability over mono‐treatment of either compound as seen in our *C. elegans* model. However, the non‐susceptibility of the sterile JK1107 mutants to FUdR and H_2_S killing (produced by bacteria in the presence of GSH or cysteine) argue against this mechanism. It seems that the combined toxicity requires the presence of germline cells. This is consistent with the fact that *C. elegans* somatic cells are post‐mitotic, meaning that the germline is the only actively dividing tissue in adult nematodes. However, it is unclear how this impacts the killing of adult animals.

In both Caco‐2 and HT‐29 cells, combined exposure to FUdR and sulphide only slightly reduced cell viability over monotreatment of either compound, although the combined treatment resulted in the greatest ATP loss. This outcome is drastically different from the results observed in reproductively active *C. elegans*, where the overwhelming outcome was rapid death.

Nevertheless, the results suggest that sulphide exposure during treatment with FUdR is an undesirable outcome that could further reduce cell proliferation, especially in rapidly dividing cells such as the epithelial layers lining the intestine, which require high turnover rates to maintain the integrity of the mucosal lining (Barker et al., 2008). It also suggests that existing genotoxicity could predispose one towards sulphide‐induced toxicity (Attene‐Ramos et al., 2006).

The gastrointestinal tract is where 10^11^ bacteria are estimated to make their home (Sender et al., 2016). Among the many members of the gut microbiome, H_2_S is known to be produced mainly by the sulphate reducing bacteria (SRB), of which the major genera comprise the *Desulfovibrio*, *Desulfobacter*, *Desulfobulbus* and *Desulfotomaculu* (Rabus et al., 2015). Other facultative anaerobes from the Enterobacteriaceae also convert cysteine as well as cysteine‐containing molecules into hydrogen sulphide through enzymatic reduction. The production of H_2_S by Enterobacteriaceae species may, in fact, be a positive feedback loop to increase Enterobacteriaceae abundance because – under inflammatory conditions – which are prevalent during chemotherapy, the production of tetrathionate from sulphide detoxification allows *Salmonella* to use it as an electron acceptor and overcome colonisation resistance (Winter et al., 2010). Our described mechanism, whereby GSH or cysteine is catabolised by Enterobacteriaceae species to produce H_2_S is another way by which H_2_S concentrations can increase in the gut.

In addition to that, bacterial adhesion to the epithelia could also be enhanced by a high H_2_S concentration produced from the catabolism of sulphur‐containing amino acids in the diet (Dordević et al., 2020). While we were not able to observe a direct increase in bacterial adhesion from GSH catabolism to H_2_S alone, the action of sufficient sulphide on the epithelia resulting in enhanced bacterial adhesion is indicative of what could happen in vivo since 2 mM of sulphide is an easily achievable level of sulphide in the intestinal lumen (Blachier et al., 2020). Furthermore, this enhancement did not require any pre‐treatment with FUdR, suggesting that otherwise healthy individuals may also experience an enhancement of bacterial adhesion in the colon with sufficient production of sulphide by the microbiota.

Although it is not currently known what the exact mechanism is by which H_2_S enhances bacterial adhesion, one possibility could be a degradation of the epithelial mucus layer by sulphide. HT‐29 cells are known to be able to produce MUC2, although some stimulation is required (Navabi et al., 2013). A report by Ijssennagger et al. (2016)) showed that the intestinal mucus layer was significantly more penetrable by bacteria in patients who harboured high levels of sulphide‐producing sulphate‐reducing bacteria as sulphide was hypothesised to reduce important disulphide bonds in the MUC2 glycoprotein that is a major component of intestinal mucus. This would render the mucus more fluid and penetrable which could allow pathobionts to gain a foothold in an otherwise impenetrable niche protected by the colonisation resistance of the pre‐existing microbiota.

We thus suggest that patients undergoing chemotherapy with 5‐FU avoid taking GSH supplements in large amounts as high antioxidant levels have led to the failure of chemotherapy by providing a huge buffer against cellular stressors (Sayin et al., 2014). Other measures could also take the form of avoiding food rich in sulphur‐containing amino acids since that could be used for catabolism to H_2_S (Singh et al., 2009) resulting in enhanced bacterial adhesion and hence, risk of infection which is undesirable in an immunosuppressed state caused by chemotherapy (Morrison, 2014). Furthermore, the presence of high H_2_S concentrations could result in intestinal mucositis, an often seen side effect in colorectal cancer patients undergoing 5‐FU therapy (Sonis et al., 2004). Mucositis could thus be potentially explained by the combined effects of an increase in bacterial adhesion by sulphide‐induced mucus layer breakdown that leads to inflammatory immune responses combined with the general inhibition of epithelial and goblet cell turnover by chemotherapy.

The gut microbiome performs numerous essential functions, yet so little is known about its interactions with the human body (Baquero & Nombela, 2012). In a diverse community of symbionts, pathobionts and commensals, the line between each can shift depending on exogenous factors. A commensal may turn pathogenic under the right conditions when the host is undergoing chemotherapy. Whether the mechanism we uncovered in *C. elegans* could be relevant in the human gut, particularly during chemotherapy of colorectal cancer patients, await further studies.

## 4 EXPERIMENTAL PROCEDURES

### 4.1 Chemicals and reagents

All reagents were purchased from Sigma‐Aldrich unless otherwise specified. GSH and GSSG are extremely acidic in water, forming a pH 4 solution at 20 mM that could cause acid stress in either *C. elegans* or bacteria and change their physiological responses (Ku & Gan, 2019). Hence, the pH of GSH and GSSH solutions used were adjusted to pH 7 with sodium hydroxide for all experiments.

### 4.2 Bacterial strains used and genetic manipulation in *E. coli* MG1655

All bacterial strains used are listed in Table 2. Strains were maintained on LB agar plates containing necessary antibiotics. Generation of *tnaA* deletion mutant was done using a λ‐RED recombination system to replace the target gene with a resistance cassette as described (Datsenko & Wanner, 2000). A PCR product containing an FRT‐flanked zeocin resistance cassette targeting ~40 nucleotides up and downstream of *tnaA* was generated and electroporated into *E. coli* MG1655 that expresses λ‐RED recombinases, Flp recombinase and SacB levansucrase on the pACYC184 plasmid containing tetracycline resistance (Chang & Cohen, 1978). Successful replacements were selected using LB plates containing 10 μg/ml zeocin. The zeocin resistance cassette was flipped out by incubating zeocin‐resistant colonies in LB containing 0.2% rhamnose which induces expression of Flp. The pACYC184 plasmid was then cured by incubating tetracycline‐resistant clones in LB containing 20% sucrose for 8–16 h and selecting colonies that were tetracycline sensitive.

**TABLE 2:** List of bacterial strains used in this study

Strain	Relevant characteristics	Reference
*Escherichia coli* OP50	*Caenorhabditis elegans* food strain	Brenner (1974)
*E. coli* MG1655	Reference K‐12 strain	Reigstad et al. (2007)
*E. coli* MG1655 Δ*tnaA*	Deletion mutant of *tnaA*	This study
*E. coli* MG1655 Δ*tnaA‐*pUCP28T	Deletion mutant of *tnaA* containing empty vector	This study
*E. coli* MG1655 Δ*tnaA* pUCP28T‐*tnaA*	Deletion mutant of *tnaA* complemented with vector carrying *tnaA*	This study
*S. enterica* Typhimurium LT2	ATCC reference strain	McClelland et al. (2001)
*A. baumanii* 17978	ATCC reference strain	Valentine et al. (2008)
*B. thailandensis* E264	ATCC reference strain	Brett et al. (1998)
*E. cloacae* 13047	ATCC reference strain	Ren et al. (2010)
*E. hormaechei* 700323	ATCC reference strain	Téné et al. (2014)
*K. pneumoniae* SGH10	Reference strain for K1/ST23 lineage	Lam et al. (2018)
*K. pneumoniae* SGH10 Δ*wcaJ*	Deletion mutant of *wcaJ*, non‐capsulated	Tan et al. (2020)
*S. aureus* 25923	ATCC reference strain	Treangen et al. (2014)

The *tnaA* mutant was complemented through electroporation with the original gene cloned into a pUCP28T plasmid vector containing a trimethoprim resistance cassette (West et al., 1994). Successful transformants were then selected on 10 μg/ml trimethoprim LB plates and confirmed using colony PCR. Primer sequences for both deletion and complementation of *tnaA* are presented in Table 3.

**TABLE 3:** List of primers used for cloning and mutant generation

Primer name	Sequence
*tnaA* KO F	ACAGGGATCACTGTAATTAAAATAAATGAAGGATTATGTAGTGTAGGCTGGAGCTGCTTC
*tnaA* KO R	GAGAGTGGCTAACATCCTTATAGCCACTCTGTAGTATTAAGTAACGCACTGAGAAGCCCT
*tnaA* clone F	CATGATTACGAATTC ATTAGATTCAATGTGATCTATTTGTTTGC
*tnaA* clone R	CTTGCATGCCTGCAG TTAAACTTCTTTAAGTTTTGCGGTGA

### 4.3 *C. elegans* maintenance, synchronisation and killing assay

Synchronised wild‐type N2 adult *C. elegans* were maintained in the S‐complete medium as described (Solis & Petrascheck, 2011) at 20°C. Synchronisation was achieved by collecting eggs, larvae and adult *C. elegans* and treatment with 25% v/v household bleach and 1 M NaOH in deionised water for 5 min, vortexing every minute until adult nematodes and larvae have dissolved. The eggs were then washed in M9 buffer and S‐complete and left to develop for 60 h into adult *C. elegans* on Nematode growth medium (NGM). Sterilisation of the adult *C. elegans* was achieved by the addition of 300 μM FUdR at this point for at least 24 h before the start of any killing assays.

To prepare GSH or GSSG for incubation with *C. elegans*, 10 μl of sterile, pH‐adjusted GSH (200 mM) or GSSG (100 mM), as well as 10 μl of bacteria (OD_600_ = 5), was added to relevant wells of a 96‐well plate containing ≥20 *C. elegans*. The final concentration of GSH or GSSG used was 20 or 10 mM in a total well volume of 100 μl. *C. elegans* death was characterised by a null response to physical stimulation as well as a rigid, rod like morphology. Other reducing agents were tested in the same way as GSH including dithiothreitol (DTT) and tris(2‐carboxyethyl)phosphine (TCEP).

### 4.4 Detection of hydrogen sulphide using lead acetate strips

In an adapted method (Livshits et al., 2017), lead acetate strips (Sigma 06728) were stuck to the side of NGM plates (Solis & Petrascheck, 2011) using adhesive tape to detect the presence of hydrogen sulphide. GSH or l‐cysteine was dissolved in deionised water at stock concentrations of 200 mM and 300 mM, respectively, and filtered through a 0.22 μm filter to ensure sterility. Each compound was then mixed with 150 μL of overnight *E. coli* MG1655 culture grown in LB to achieve final concentrations of 20 and 3 mM of GSH and l‐cysteine, respectively. The culture was then spread evenly across the nutrient agar plate, sealed with Parafilm and incubated at 20°C for 24 h. The lead acetate strips remained white if no sulphide was produced or turned black due to the formation of lead (II) sulphide indicating the presence of hydrogen sulphide.

### 4.5 Measurement of sulphide production by *E. coli* MG1655 with GSH or cysteine over 24 h

A methylene blue absorbance assay (Livshits et al., 2017) was used to measure sulphide concentrations by capturing sulphide in a 0.85% zinc acetate/3% sodium hydroxide capture solution dissolved in deionised water. To 100 μL of capture solution from each replicate, 33.3 μl each of 20 mM N,N‐dimethyl‐*p*‐phenylenediammonium and 28 mM stock of iron (III) chloride dissolved in 7.2 and 1.2 M hydrochloric acid, respectively, was then added and mixed thoroughly. The absorbance of samples was read at 670 nm using a Synergy H1 microplate reader (Biotek) and compared to a sulphide standard curve generated using 0 to 1.5 mM of Na_2_S (MP Biomedicals) dissolved in water. For each treatment replicate, an overnight culture of *E. coli* MG1655 bacteria grown in 15 ml of LB was prepared. The culture was concentrated 150‐fold by volume and 150 μl was placed in the centre of a 3 cm diameter petri dish. To prepare GSH or l‐cysteine, each compound was dissolved in water and filtered through a 0.22 μm filter to achieve stock concentrations of 200 mM and 300 mM, respectively. Each compound was then mixed with the bacterial culture to achieve final concentrations of 20 mM and 3 mM of GSH and l‐cysteine, respectively. The final volume of bacteria‐GSH/l‐cysteine suspension was made up to 800 μl with sterile PBS. The 3 cm dish was in turn placed inside another 6 cm diameter petri dish filled with 4 ml of zinc acetate/NaOH capture solution. The plates were then sealed using Parafilm to minimise loss of H_2_S until measurement of sulphide concentrations at each timepoint.

### 4.6 Cellular XTT viability assays

Caco‐2 and HT‐29 MTX P8 colorectal adenocarcinoma cells were maintained at 37°C in complete Dulbecco’s Modified Eagle Medium (DMEM) (Gibco) supplemented with penicillin/streptomycin, non‐essential amino acids (Sigma‐Aldrich) and 10% foetal bovine serum (Biowest) in a 5% CO_2_ humidified incubator (Panasonic). For FUdR pre‐treatment, 50 μM of FUdR was added for 24 h prior to the start of the assay and removed by washing the cells with PBS. Exposure to sulphide was achieved by adding 3 mM sodium sulphide (Na_2_S) as a sulphide donor in the solution. Samples were treated with sulphide two times 2 h apart for a total duration of 4 h and then washed with PBS before the addition of fresh DMEM media for XTT viability assays. XTT assays were carried out according to the manufacturer’s instructions (Biotium) and samples were read using a Synergy H1 microplate reader (Biotek) at 475 nm and subtracting background at 660 nm.

### 4.7 Analysis of intracellular ATP concentrations

HT‐29 MTX P8 cells were cultured in complete DMEM at a density of 10^5^ cells/well for 3 days with 10 μM of (N‐[N‐(3,5‐difluorophenacetyl)‐l‐alanyl]‐*S*‐phenylglycine *t*‐butyl ester (DAPT), a γ‐secretase inhibitor that promotes goblet cell maturation responsible for mucus production (Sigma) as described (Navabi et al., 2013). Prior to infection, the media was changed to DMEM containing 10% FBS without any antibiotics. For FUdR treatment, 100 μM of FUdR was added for 24 h prior to bacterial inoculation. The FUdR‐containing media was removed and fresh infection DMEM was added for bacterial infection. Overnight bacterial culture grown in LB was then added at an MOI of 50. ATP was then extracted from cells after 4 h of incubation with bacteria with or without 10 mM of GSH. The cells were then washed twice with PBS before detachment using 200 μl trypsin–EDTA for 8 min followed by the addition of 800 μL of DMEM containing 10% FBS. Following this, the cells were pelleted by centrifugation at 300*g* for 5 min and subjected to further processing as described (Yang et al., 2002). The lysate was then used for ATP analysis using the ENLITEN® ATP Assay System from Promega following kit instructions.

### 4.8 Evaluation of bacterial adhesion to HT‐29 MTX P8 cells

HT‐29 MTX P8 cells were cultured in complete DMEM at a density of 10^5^ cells/well in a 24‐well plate for 3 days supplemented with 10 μM DAPT. The cell culture media was then switched to DMEM containing no antibiotics for 24 h before relevant bacterial strains were added at an MOI of 50. GSH that was adjusted to pH 7 with 5 M NaOH or Na_2_S were then added to relevant wells. The plate was centrifuged for 5 min at 250*g* to maximise bacteria‐cell contact and incubated in a 5% CO_2_ humidified incubator at 37 °C for 2 h. After 2 h, monolayers were washed twice with PBS and the cells were lysed using 1 ml 0.25% Triton‐X in PBS. The lysate was then diluted and 100 μl plated on LB agar at the appropriate dilutions to enumerate bacterial CFU.

### 4.9 Statistical analyses

Student’s unpaired *t*‐test was conducted to compare the means between two groups. One way ANOVA followed by Tukey’s or Dunnett’s post hoc tests were conducted for comparisons of the means between three or more groups. All statistical tests were conducted using the GraphPad Prism software. A two‐tail *p*‐value of <.05 is considered statistically significant.

## CONFLICT OF INTEREST

The authors declare no conflict of interest.

## AUTHOR CONTRIBUTIONS

YHG conceived the study. YHG, YC, DL designed the study. DL, LFN, YC performed the experiments, all authors are involved in the acquisition, analysis, or interpretation of the data; and YHG and DL wrote the manuscript with input from all authors.

## ETHICS APPROVAL STATEMENT

The studies described obtained are exempted from ethics approval by Institutional Review Board as no animals or human samples were used, except cell‐lines.

## Supporting information


Figure S1‐S5

## Data Availability

The data that supports the findings of this study are available in the supplementary material of this article
