# Supplementary material for: Glutathione catabolism by Enterobacteriaceae species to hydrogen sulphide adversely affects the viability of host systems in the presence of 5′fluorodeoxyuridine
Source: Mol Microbiol. 2022 Mar 22;117(5):1089–103. doi: 10.1111/mmi.14893 (PMC9313583; doi:10.1111/mmi.14893)
Supplement: Supplementary file 1 — Figure S1‐S5 [file MMI-117-1089-s001.docx]

List of Supplementary Figures:

1. S1: Dose-response curve of *C. elegans* killing to GSH with overnight or log-phase bacterial cultures
2. S2: Response of sterile *C. elegans* to FUDR and bacteria with GSH
3. S3: Effects of various reducing agents on FUDR-induced bacterial rapid killing of *C. elegans*
4. S4: Cell counts of HT-29 MTX P8 colorectal adenocarcinoma cells
5. S5: Bacterial growth curves in the presence of GSH or sulfide


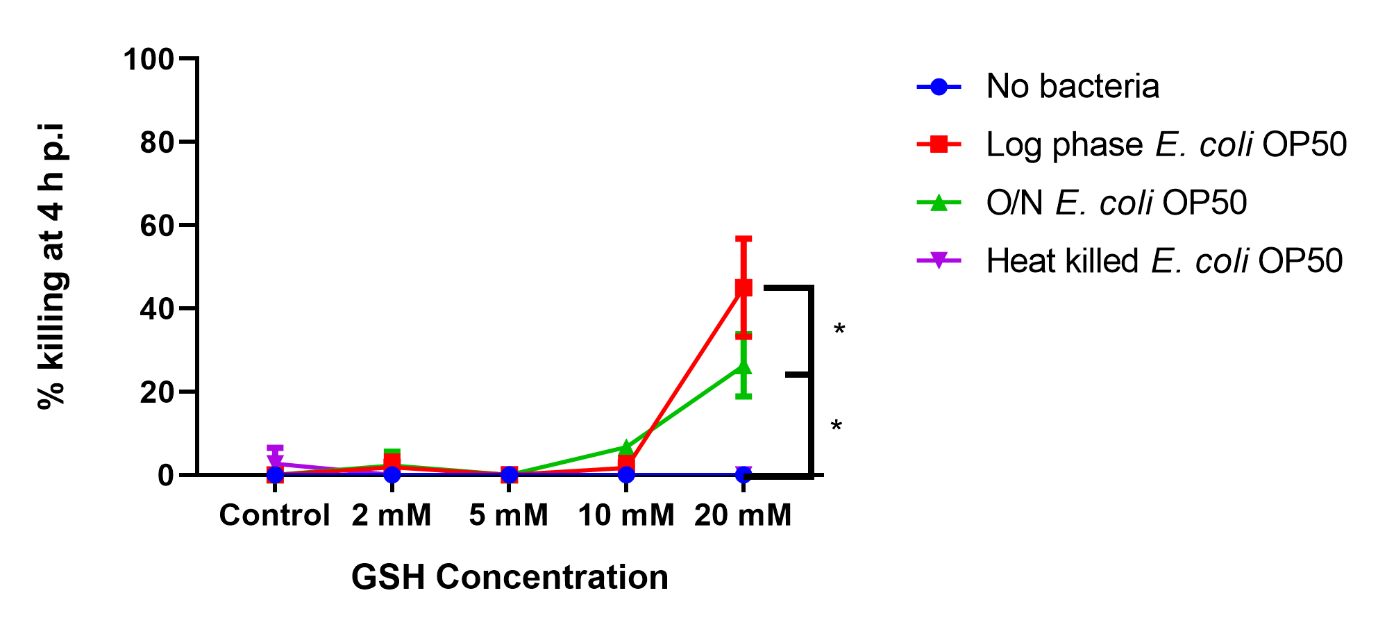


**S1.** Dose-response curve of *C. elegans* killing when exposed to increasing concentrations of GSH with overnight (O/N), log phase, heat-killed *E. coli* OP50 or medium (no bacteria) for 8 hours. Means ± SD are plotted (n=3). Student’s t-test p-values comparing treatment groups to the uninfected control are shown. * p<0.05.

**S2** **(A)** Adult wild-type N2 (WT) and temperature-sensitive sterile JK1107*C. elegans* were incubated with *E. coli* MG1655 in the presence or absence of GSH for 8 h. A horizontal line represents means of each treatment group ± S.D (n=9 from three separate experiments). Student’s t-test was used to compare the GSH treatment group to the no GSH control. Ns = not significant.

**S2 (B)** Adult wild-type N2 (WT) and temperature sensitive sterile JK1107*C. elegans* were pre-incubated with 300 µM of FUdR for 24 h. Subsequently, the worms were incubated with *E. coli* MG1655 in the presence or absence of GSH for 8 h. A horizontal line represents means of each treatment group ± S.D (n=9 from three separate experiments). Student’s t-test was used to compare the GSH treatment group to the no GSH control. Ns = not significant. *** p<0.001.


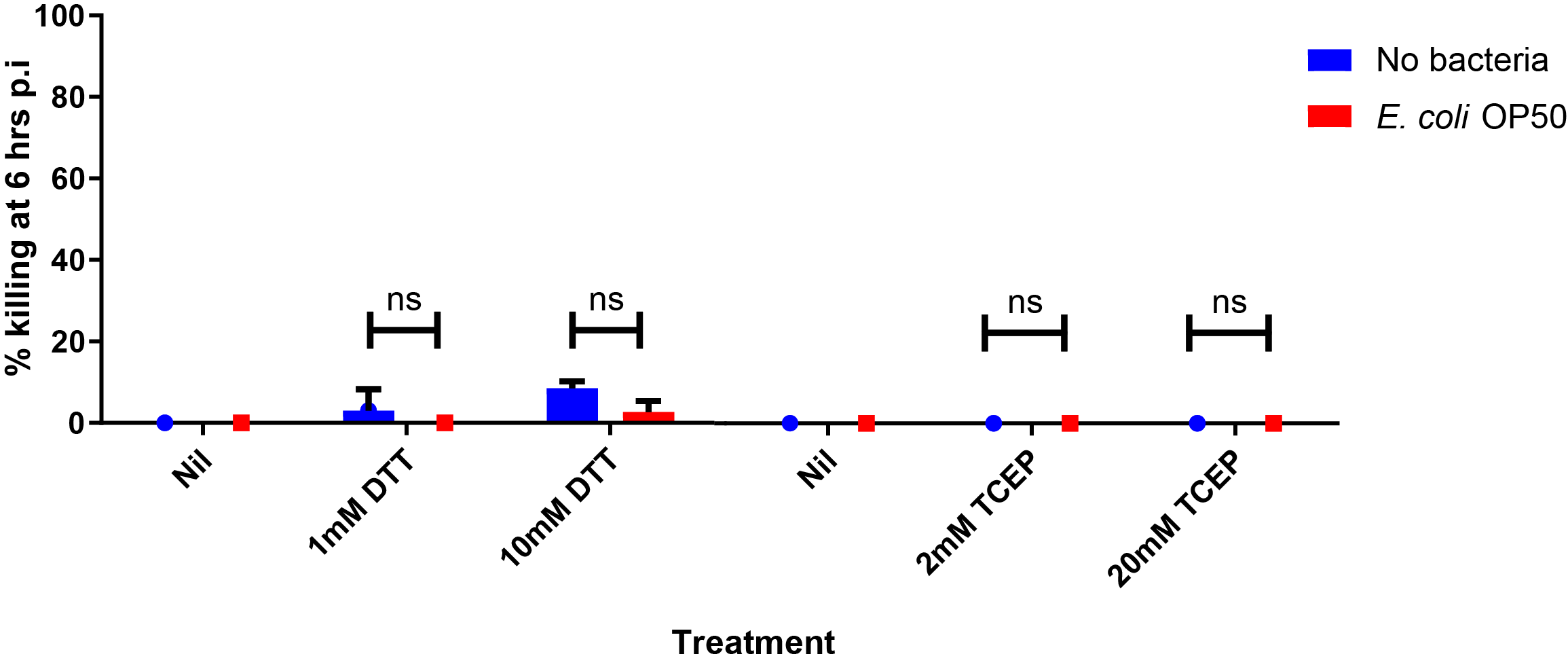


**S3**. Killing of *C. elegans* incubated with *E. coli* OP50 and two different types of reducing agents. Reducing agents both thiol-based DTT (similar to GSH) and non-thiol-based (TCEP) did not induce any appreciable killing over untreated controls. Means ± SD are plotted (n=3). Student’s t-test was used to compare the OP50 with DTT or TCEP treatment group to the no bacteria control. Ns = not significant.


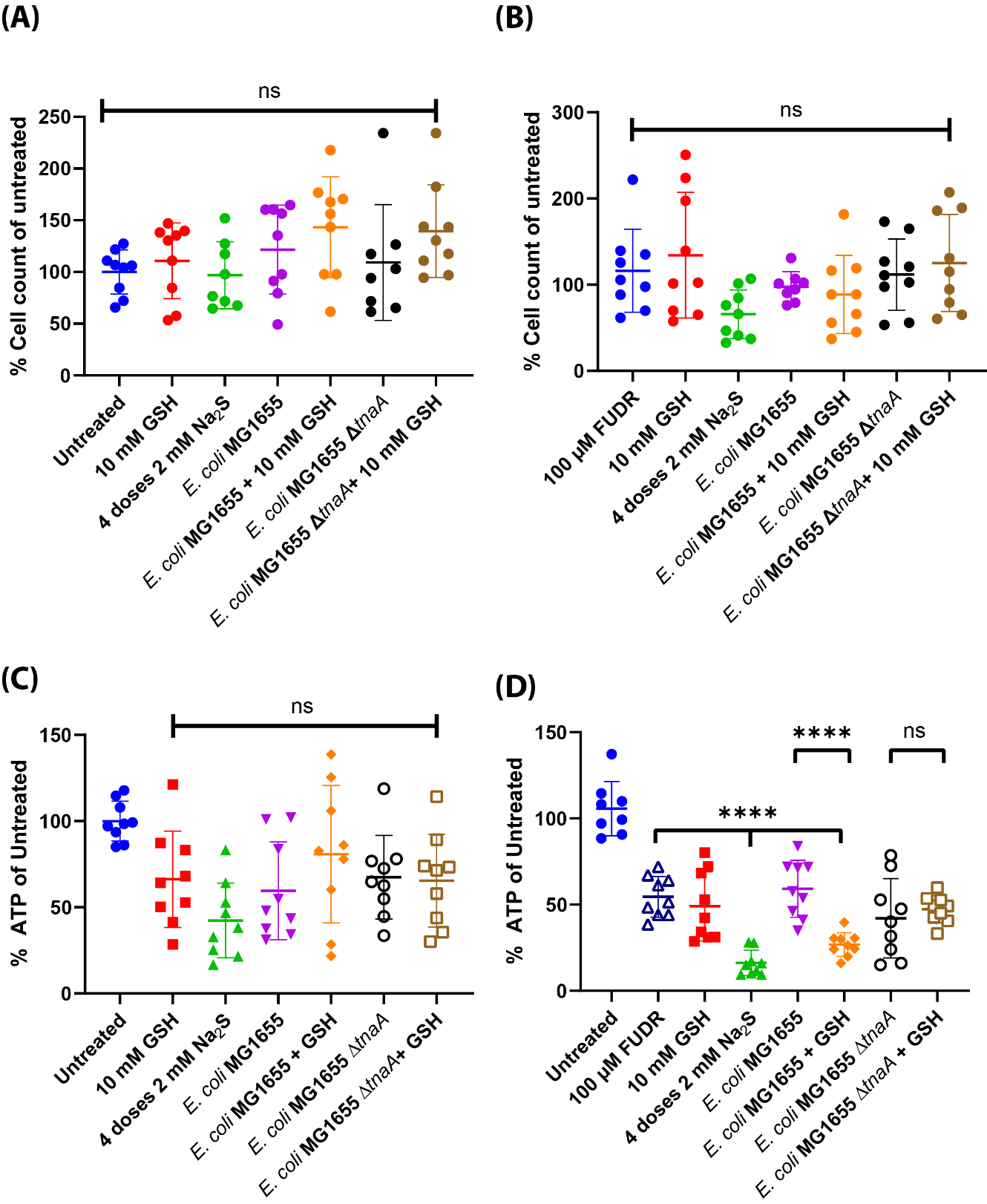


**S4** Cell counts for HT-29 MTX P8

(A) Cell counts for 2x10^5^ HT-29 MTX P8 cells treated with 10 µM DAPT for 3 days and subsequently with 10 mM GSH, 4 doses of 2 mM Na_2_S, MOI 50 of *E. coli* MG1655 with and without 10 mM GSH or *E. coli* MG1655 Δ*tnaA* with or without 10 mM of GSH added are shown. (B) Cell counts for 2x10^5^ HT-29 MTX P8 cells treated with 10 µM DAPT for 3 days and 100 µM FUDR for 24 h prior to treatment with 10 mM GSH, 4 doses of 2 mM Na_2_S, *E. coli* MG1655 with and without 10 mM GSH or *E. coli* MG1655 Δ*tnaA* with or without 10 mM of GSH added. One-way ANOVA was used to compare the means within each treatment group. ns = not significant.


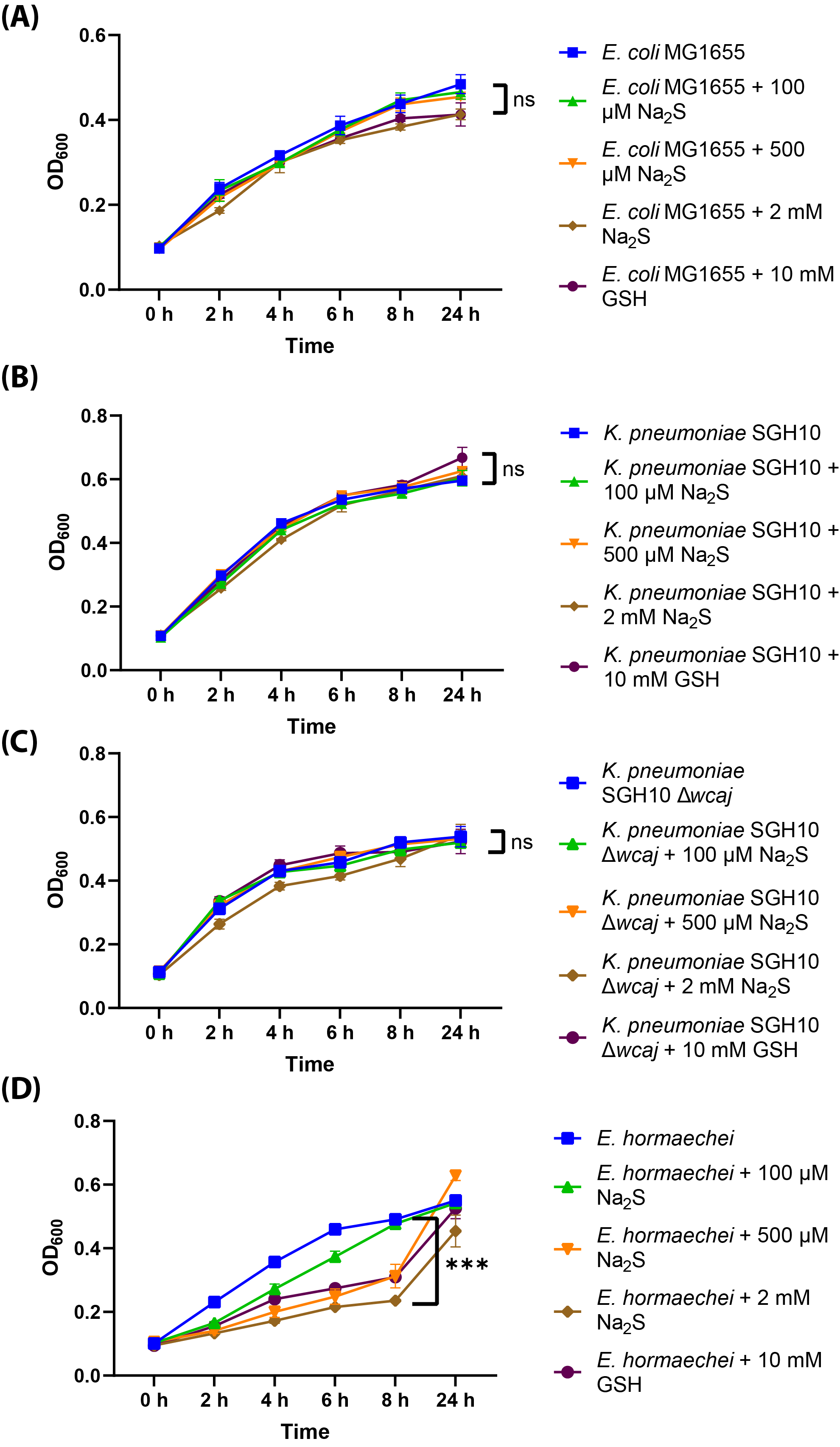


**S5**. Growth curves of various bacterial species in the presence of GSH or Na_2_S. Sulfide concentrations from 100 µM to 2 mM were incubated with bacteria in LB media and OD_600_ readings measured every 2 hours. Means ± SD are plotted (n=3) One-way ANOVA was used to compare bacteria only control to treatment groups after confirming that data followed a normal distribution using Shapiro-Wilk test. Dunnett post-hoc multiple comparison was then used to find significantly different groups. ***=p<0.001. ns. denotes not significant.

**Supplementary methods:**

**Examining GSH dose-response on *C. elegans* with log-phase or overnight bacteria**

Synchronised, FUDR pre-treated *C. elegans* in 80 µL S-complete medium were incubated with 10 µL of bacterial cells (OD_600_ 5) from overnight culture (grown in LB broth for about 16 hours), log-phase culture (grown in LB broth for 4 hours), heat-killed culture (log-phase culture boiled for 5 minutes) or no bacteria (S-complete medium). Then 10 µL of sterile-filtered GSH solutions were added at increasing concentrations from 2 to 20 mM. *C. elegans* death was scored after 8 hours at 20 °C. The death was characterised by a null response to physical stimulation as well as a rigid, rod like morphology.

***C. elegans* killing assay with DTT and TCEP**

DTT and TCEP were prepared by dissolving each compound in deionised water and then sterile-filtered. Synchronised, FUDR pre-treated *C. elegans* in 80 µL S-complete medium were then incubated with 10 µL of overnight culture of bacteria (OD_600_ 5 in S-complete medium) and 10 µL of either DTT or TCEP at 20 °C. After 8 hours, *C. elegans* death was enumerated. *C. elegans* death was characterised by a null response to physical stimulation as well as a rigid, rod like morphology

**Bacterial growth curves**

Overnight bacterial culture was prepared in LB and diluted 10-fold into a total reaction volume of 200 µL LB in a 96-well plate. GSH and Na_2_S stock solutions were prepared at concentrations of 100 mM and 200 mM respectively and diluted accordingly into the LB culture. Growth was measured by using a microplate reader to measure OD_600_ of the culture at 2, 4, 6, 8 and 24 h post-incubation.

**Cell viability counting using trypan blue**

HT-29 MTX P8 cells were cultured in complete Dulbecco's Modified Eagle (DMEM) at a density of 10^5^ cells/well for 3 days with 10 µM of (N-[N-(3,5-difluorophenacetyl)-L-alanyl]-S-phenylglycine t-butyl ester (DAPT), a γ-secretase inhibitor that promotes goblet cell maturation responsible for mucus production (Sigma) as used by (Navabi et al., 2013). Prior to infection, the media was changed to DMEM containing 10% FBS without any antibiotics. For FUDR treatment, 100 µM of FUDR was added for 24 hours prior to bacterial inoculation. The FUDR-containing media was removed and fresh infection DMEM added for bacterial infection. Overnight bacterial culture grown in LB was then added at a MOI of 50. After 4 h, the culture media was removed, and the cell monolayer washed with PBS before addition of 200 µL of trypsin-EDTA for 6 minutes to detach adherent cells for enumeration. 800 µL of DMEM was then added to neutralise the trypsin-EDTA solution. 0.4% Trypan blue in PBS was then added in a 1:1 ratio with the cell culture and 10 µL of the resulting suspension was then enumerated for cell counts using a haemocytometer.
